# The Treatment of Diabetes

**Published:** 1890-08-09

**Authors:** 


### The Practitioner's Mirror and Retrospect.

*[The Editor will be glad to receive offers of co-operation and contributions from members of the profession. There is no doubt that much valuable material full of interest and instruction is at present lost to science. This is largely so in the case of* (1) *the younger and less-known members of the hospital staff* (2) *those connected with cottage hospitals, and* (3) *the great body of medical practitioners not attached to any institution. The co-operation of all is cordially invited to enable the Editor to make* The Hospital *of the utmost possible use and value to the profession. Specialists willing to review books on their special subjects are invited to communicate this fact at once. All letters should be addressed to* The Editor, The Lodge, Porchester Square, London, W.]

### MEDICINE.

### TREATMENT OF DIABETES.

The name Glycosuria, from the presence of glucose in the urine, is certainly to be preferred to that of diabetes, which, like diarrhœa, merely refers to the excessive flow of urine, a phenomenon neither constant nor distinctive, since there are other totally different conditions, neuroses, not derangements of assimilation, in which there is an inordinate elimination of water by the kidneys; and to designate these respectively as diabetes mellitus, and insipidus suggests a relation that has no real existence. But at the same time, though clinical experience shows that as a rule the patient's general health may be very fairly maintained so long as the elimination of sugar is kept at a low point, while it rapidly deteriorates with or in consequence of an increase of the sugar in the urine, whether this be the result of the ingestion of carbohydrates or not; some physicians are of opinion that too much weight has been attached to this particular symptom. The dietaries recommended by different authorities have varied accordingly within certain limits, the advocates of a "strict" diet, or one consisting exclusively of albumen and fats, being represented by Cantani, who practically excludes everything else, and the "liberal" school by A. von Dühring, who even permits white bread *ad lib.* for breakfast. Donkin pointed out that milk sugar is very fairly metabolised by most patients, and Külze that fruit sugar (levulose) and inulin, may be permitted with like impunity.

At the recent congress of the *Verein für innere Medicin*, Dr. Jaques Meyer reviewed the whole question, and, while admitting that it was quite possible to keep the excretion of sugar at a minimum by a strict diet, he expressed a doubt whether other dangers might not be induced by the ingestion of an excessive amount of nitrogenous matters, the products of the metabolism of which were uric acid, creatin, creatinin, &c., which, if retained in the system, gave rise to the conditions known as uræmia. He referred to the opinion of Naunyn that the coma which so frequently ushers in the fatal termination in diabetes was of a uræmic nature, due not to any impaired excretory power in the kidneys themselves, but to the accumulation in the system of these waste products of imperfect metabolism, and he observed that sudden death was more frequent in this than in any other chronic disease, being, in fact, as he suggested, the consequence of the dietetic treatment employed. He had not seldom seen albuminuria to be generally preceded by an excessive excretion of urates and oxalates of calcium, all these abnormal conditions indicating imperfect albuminous metabolism. Even Cantani admitted this, though he looked on oxaluria, and possibly albuminuria, in such cases as pathological conditions of an origin akin to the glycosuria, and not as the natural physiological results of the ingestion of an excess of albumin above the powers of the organism to assimilate and metabolise. Glück had strongly insisted on the existence of a psychic factor, as in a case in which under careful diet the urine had become completely free from sugar, a strong emotional disturbance was followed by the appearance of no less than 7 per cent., though no carbohydrates had been taken.

Meyer was inclined to deprecate the too exclusive and excessive administration of flesh in a disease in which the functions of digestion were notoriously impaired; some diabetics, as Hirschfeld had pointed out, could not easily assimilate either flesh or fat, and Leyden and Rosenstein had found atrophy of the peptic glands. He himself allowed not only milk, cream, butter, fat, cheese, and eggs, but in the earlier stages of the disease 80 to 120 grammes of carbohydrates daily.

When the general nutrition was impaired he was in the habit of allowing the patient to adopt a mixed diet, with reasonable moderation in carbohydrates, resuming the strict diet every sixth week or thereabouts; but, especially when meat was not well borne, or oxaluria or renal concretions were observed, giving from one to two litres of milk.

In fact, he was convinced that an exclusive and excessive flesh diet might aggravate the danger, superadding that of uræmia, which, rather than the primary disease, was a frequent cause of death. Death, indeed, from this cause not seldom occurred suddenly and unexpectedly, when the urine was free from sugar, or nearly so.

As to drugs, he relied on ordinary tonics—quinine and iron—but found marked benefit in many cases from arsenic, under which he had seen an increase of body weight to the extent of 8 kgs., even though sugar was still present in the urine.

Meyer comes to the following conclusions :—

1. That the daily excretion of sugar is not an absolute indication either of the intensity or of the development of the disease.

2. That there do not exist several forms of the disease, the only differences being those of the acute, subacute, and chronic.

3. A moderately mixed diet is on the whole preferable to one consisting too exclusively of flesh, since this may give rise to uræmic poisoning.

4. That the subjective need for food, that is to say, the craving appetite, must not be indulged, being far beyond the capabilities of the organism for digestion and normal metabolism, and that a few good meals are better than frequent lighter ones.

5. That moral treatment is important.

6. Diabetes is only in rare cases curable.

7. That the cardiac disease has a common origin and is due to arterial selerosis.

